# Defining laboratory medicine, or squaring the circle?

**DOI:** 10.11613/BM.2021.010402

**Published:** 2021-02-15

**Authors:** Joseph Watine

**Affiliations:** Laboratoire de biologie polyvalente, Hôpital général, Villefranche-de-Rouergue, France

**Keywords:** laboratory medicine, education, editorial practice, research methodology, writing in science

## Abstract

In the August 2020 issue of Clinical Chemistry and Laboratory Medicine, Giuseppe Lippi and Mario Plebani proposed a definition of laboratory medicine, which ends with this sentence: “The results of these measurements are translated into actionable information for improving the care and/or maintaining the wellness of both a single individual and an entire population”. Nevertheless, the selfishness of individuals may, sometimes, jeopardize the interest of whole populations. The virtue of justice being within the reach of the entire human community more than of single individuals, the final sentence in the definition proposed by Giuseppe Lippi and Mario Plebani, should therefore, in our view, be rewritten, less selfishly, for example like this: “For a given investment, these measurements are preferably made when they bring as much beneficence, and non-maleficence, as possible to the whole population”.

In the August 2020 issue of Clinical Chemistry and Laboratory Medicine, Giuseppe Lippi and Mario Plebani proposed what they called a “pragmatic” definition of laboratory medicine, which ends with this sentence: “The results of these measurements are translated into actionable information for improving the care and/or maintaining the wellness of both a single individual and an entire population.” (*1*). The authors also wrote that this definition “may be seen as a good starting point for widespread discussion and/or endorsement”, which sounds like an invitation to respond. I am responding below.

The selfishness of individuals may, sometimes, jeopardize the interest of whole populations. Therefore, sometimes, satisfying “both a single individual and an entire population” is likely to look like an attempt to square the circle.

For example, individual doctors may recommend prostate cancer screening with annual prostatic specific antigen (PSA) blood test over the age of 50 years. They believe that this allows less aggressive treatments of cancers diagnosed earlier. Governmental organizations generally advise against that, and rather emphasize: 1) frequently elevated PSA without cancer, causing unnecessary, or even harmful, explorations, bearing in mind that screening is aimed at asymptomatic patients; 2) over-diagnosis, that is the diagnosis of cancers which would never have led to a disease, with all the adverse consequences of curative treatments, including incontinence and impotence; and 3) a lack of effect of screening on all-cause mortality.

This example illustrates that, individuals, faced with the same evidence, may end up with diametrically opposed decisions, thus ensuring their autonomy, which is a core bioethical value (*2*).

Autonomy mainly concerns individuals. Equity, which is another core bioethical value, mainly concerns the human community (*2*). Equity (or justice) is a virtue which consists in managing one’s conduct on the natural sentiment of the fair and of the unfair (*2*, *3*). In our example of prostate cancer screening, is it fair to waste massive resources worldwide, to reach a doubtful benefit-to-harm ratio, whereas the same resources could avoid truer misfortunes, for example in providing access to drinking water to entire populations who are deprived of it?

Hence this proposal of rewriting the final sentence in the definition proposed by Giuseppe Lippi and Mario Plebani, using a less individual formulation, for example: “For a given investment, these measurements are preferably made when they bring as much beneficence, and non-maleficence, as possible to the whole population”.
